# Supplementary material for: Composition and functional diversity of soil and water microbial communities in the rice-crab symbiosis system
Source: PLoS One. 2025 Jan 17;20(1):e0316815. doi: 10.1371/journal.pone.0316815 (PMC11741653; doi:10.1371/journal.pone.0316815)
Supplement: Attachment — Submitted filename: PONE-D-24-35167.pdf [file pone.0316815.s002.pdf]

PLOS ONE

Comparative Analysis of Soil and Water Microbial Community Structures and Functions in the Rice-Crab Symbiotic System Based on High-throughput Sequencing

--Manuscript Draft--

**Manuscript Number:**	PONE-D-24-35167
**Article Type:**	Research Article
**Full Title:**	Comparative Analysis of Soil and Water Microbial Community Structures and Functions in the Rice-Crab Symbiotic System Based on High-throughput Sequencing
**Short Title:**	Analysis of soil and water microorganisms in rice crab symbiosis system based on high throughput sequencing
**Corresponding Author:**	Guo Yang<br>Ningxia University<br>yinchuan, CHINA
**Keywords:**	rice and crab co-culture; microbial diversity; high-throughput sequencing; water column; substrate
**Abstract:**	Rice-crab co-culture is an environmentally friendly agricultural and aquaculture technology with high economic and ecological value. In order to clarify the structure and function of soil and water microbial communities in the rice-crab symbiosis system, the standard rice-crab field with a ring groove was used as the research object. High-throughput sequencing was performed with rice field water samples to analyze the species and abundance differences of soil bacteria and fungi. The results showed that the OTU richness and community diversity in soil were significantly higher than those in water, while there were significant differences in soil microbial diversity and OTU richness in water sediments.The dominant species at the bacterial phylum level were Amoebacteria, Cyanobacteria, Actinomycetes, Synechococcus and Greenbacteria, and at the genus level the dominant species were norank_f_norank_o_Chloroplast, unclassified_f_Rhodobacteraceae, LD29, Cyanobium_PCC-6307, and norank_f_MWH-UniP1_aquatic_group. The dominant species at the fungal phylum level are unclassified_k_Fungi, Ascomycota, Rozellomycota, Phaeomycota and Stenotrophomonas, and at the genus level the dominant species are unclassified_k_Fungi, unclassified_p_Rozellomycota, Metschnikowia, Cladosporium, unclassified_p_Chytridiomycota. The dominant phylum may rely on mechanisms such as organic matter catabolism, secretion of secondary metabolites and phototrophic autotrophy, as predicted by functional gene analysis. The main functional genes were all related to metabolic functions, such as secondary product metabolism, energy metabolism, amino acid metabolism, etc.
**Order of Authors:**	Guo Yang
	Tian jun Cang
	wang zhi
**Additional Information:**	
**Question**	**Response**
**Financial Disclosure**<br><br>Enter a financial disclosure statement that describes the sources of funding for the work included in this submission. Review the submission guidelines for detailed requirements. View published research articles from *PLOS ONE* for specific examples.	Yes

Powered by Editorial Manager® and ProduXion Manager® from Aries Systems Corporation

This statement is required for submission and **will appear in the published article** if the submission is accepted. Please make sure it is accurate.<br><br>**Funded studies**<br>Enter a statement with the following details:<br>• Initials of the authors who received each award<br>• Grant numbers awarded to each author<br>• The full name of each funder<br>• URL of each funder website<br>• Did the sponsors or funders play any role in the study design, data collection and analysis, decision to publish, or preparation of the manuscript?<br><br>Did you receive funding for this work?	
Please add funding details.<br>as follow-up to "**Financial Disclosure**<br><br>Enter a financial disclosure statement that describes the sources of funding for the work included in this submission. Review the submission guidelines for detailed requirements. View published research articles from *PLOS ONE* for specific examples.<br><br>This statement is required for submission and **will appear in the published article** if the submission is accepted. Please make sure it is accurate.<br><br>**Funded studies**<br>Enter a statement with the following details:<br>• Initials of the authors who received each award<br>• Grant numbers awarded to each author<br>• The full name of each funder<br>• URL of each funder website<br>• Did the sponsors or funders play any role in the study design, data collection and analysis, decision to publish, or preparation of the manuscript?<br><br>Did you receive funding for this work?"	National Key Research and Development Project (No. 2021YFD1900605-04)<br>First-class discipline of Ningxia High Education Institutions (Water Engineering Discipline) funded project (Grant No. NXYLXK2021A03).

Powered by Editorial Manager® and ProduXion Manager® from Aries Systems Corporation

Please select the country of your main research funder (please select carefully as in some cases this is used in fee calculation).<br>as follow-up to "**Financial Disclosure**<br><br>Enter a financial disclosure statement that describes the sources of funding for the work included in this submission. Review the submission guidelines for detailed requirements. View published research articles from *PLOS ONE* for specific examples.<br><br>This statement is required for submission and **will appear in the published article** if the submission is accepted. Please make sure it is accurate.<br><br>**Funded studies**<br>Enter a statement with the following details:<br>• Initials of the authors who received each award<br>• Grant numbers awarded to each author<br>• The full name of each funder<br>• URL of each funder website<br>• Did the sponsors or funders play any role in the study design, data collection and analysis, decision to publish, or preparation of the manuscript?<br><br>Did you receive funding for this work?"	CHINA - CN
**Competing Interests**<br><br>Use the instructions below to enter a competing interest statement for this submission. On behalf of all authors, disclose any competing interests that could be perceived to bias this work—acknowledging all financial support and any other relevant financial or non-financial competing interests.<br><br>This statement is required for submission and **will appear in the published article** if the submission is accepted. Please make sure it is accurate and that any funding	NO

sources listed in your Funding Information later in the submission form are also declared in your Financial Disclosure statement.<br><br>View published research articles from *PLOS ONE* for specific examples.<br><br>**NO authors have competing interests**<br><br>Enter: *The authors have declared that no competing interests exist.*<br><br>**Authors with competing interests**<br><br>Enter competing interest details beginning with this statement:<br><br>*I have read the journal's policy and the authors of this manuscript have the following competing interests: [insert competing interests here]*<br><br>* typeset	
**Ethics Statement**<br><br>Enter an ethics statement for this submission. This statement is required if the study involved:<br><br>• Human participants<br>• Human specimens or tissue<br>• Vertebrate animals or cephalopods<br>• Vertebrate embryos or tissues<br>• Field research<br><br>Write "N/A" if the submission does not require an ethics statement.<br><br>General guidance is provided below. Consult the submission guidelines for detailed instructions. **Make sure that all information entered here is included in the Methods section of the manuscript.**	N/A

**Format for specific study types**<br><br>**Human Subject Research (involving human participants and/or tissue)**<br>• Give the name of the institutional review board or ethics committee that approved the study<br>• Include the approval number and/or a statement indicating approval of this research<br>• Indicate the form of consent obtained (written/oral) or the reason that consent was not obtained (e.g. the data were analyzed anonymously)<br><br>**Animal Research (involving vertebrate animals, embryos or tissues)**<br>• Provide the name of the Institutional Animal Care and Use Committee (IACUC) or other relevant ethics board that reviewed the study protocol, and indicate whether they approved this research or granted a formal waiver of ethical approval<br>• Include an approval number if one was obtained<br>• If the study involved *non-human primates*, add *additional details* about animal welfare and steps taken to ameliorate suffering<br>• If anesthesia, euthanasia, or any kind of animal sacrifice is part of the study, include briefly which substances and/or methods were applied<br><br>**Field Research**<br><br>Include the following details if this study involves the collection of plant, animal, or other materials from a natural setting:<br>• Field permit number<br>• Name of the institution or relevant body that granted permission	
**Data Availability**<br><br>Authors are required to make all data underlying the findings described fully available, without restriction, and from the time of publication. PLOS allows rare exceptions to address legal and ethical concerns. See the PLOS Data Policy and FAQ for detailed information.	No - some restrictions will apply

Powered by Editorial Manager® and ProduXion Manager® from Aries Systems Corporation

A Data Availability Statement describing where the data can be found is required at submission. Your answers to this question constitute the Data Availability Statement and **will be published in the article**, if accepted.<br><br>**Important:** Stating 'data available on request from the author' is not sufficient. If your data are only available upon request, select 'No' for the first question and explain your exceptional situation in the text box.<br><br>Do the authors confirm that all data underlying the findings described in their manuscript are fully available without restriction?	
**Describe where the data may be found in full sentences. If you are copying our sample text, replace any instances of XXX with the appropriate details.**<br><br>• If the data are **held or will be held in a public repository**, include URLs, accession numbers or DOIs. If this information will only be available after acceptance, indicate this by ticking the box below. For example: *All XXX files are available from the XXX database (accession number(s) XXX, XXX.).*<br>• If the data are all contained **within the manuscript and/or Supporting Information files**, enter the following: *All relevant data are within the manuscript and its Supporting Information files.*<br>• If neither of these applies but you are able to provide **details of access elsewhere**, with or without limitations, please do so. For example:<br><br>*Data cannot be shared publicly because of [XXX]. Data are available from the XXX Institutional Data Access / Ethics Committee (contact via XXX) for researchers who meet the criteria for access to confidential data.*<br><br>*The data underlying the results presented in the study are available from (include the name of the third party*	All relevant data are within the manuscript and its Supporting Information files

and contact information or URL).<br>• This text is appropriate if the data are owned by a third party and authors do not have permission to share the data.<br><br>* typeset	
Additional data availability information:	

Powered by Editorial Manager® and ProduXion Manager® from Aries Systems Corporation

手稿 Click here to access/download;Manuscript;guoyang1.2.5(picture)-0814.docx

Comparative Analysis of Soil and Water Microbial Community Structures and Functions in the Rice-Crab Symbiotic System Based on High-throughput Sequencing

GUO Yang[1], TIAN Juncang[1,2,3*], Wang Zhi[4]

1. Institute of Civil Engineering and Water Conservancy Engineering, Ningxia University; 2. Ningxia Water Saving Irrigation and Water Resources Control Engineering Technology Research Center; 3. Engineering Research Center of Ministry of Education of Modern Agricultural Water Resources Utilization in Dry Area; 4. Department of Earth and Environmental Sciences, California State University, CA 93740, USA

Abstract: Rice-crab co-culture is an environmentally friendly agricultural and aquaculture technology with high economic and ecological value. In order to clarify the structure and function of soil and water microbial communities in the rice-crab symbiosis system, the standard rice-crab field with a ring groove was used as the research object. High-throughput sequencing was performed with rice field water samples to analyze the species and abundance differences of soil bacteria and fungi. The results showed that the OTU richness and community diversity in soil were significantly higher than those in water, while there were significant differences in soil microbial diversity and OTU richness in water sediments.The dominant species at the bacterial phylum level were Amoebacteria, Cyanobacteria, Actinomycetes, Synechococcus and Greenbacteria, and at the genus level the dominant species were *norank_f_norank_o_Chloroplast*, *unclassified_f_Rhodobacteraceae*, *LD29*, *Cyanobium_PCC-6307*, and *norank_f_MWH-UniP1_aquatic_group*. The dominant species at the fungal phylum level are unclassified_k_Fungi, Ascomycota, Rozellomycota, Phaeomycota and Stenotrophomonas, and at the genus level the dominant species are *unclassified_k_Fungi*, *unclassified_p_Rozellomycota*, *Metschnikowia*, *Cladosporium*, *unclassified_p_Chytridiomycota*. The dominant phylum may rely on mechanisms such as organic matter catabolism, secretion of secondary metabolites and phototrophic autotrophy, as predicted by functional gene analysis. The main functional genes were all related to metabolic functions, such as secondary product metabolism, energy metabolism, amino acid metabolism, etc.

Keywords: rice and crab co-culture; microbial diversity; high-throughput sequencing; water column; substrate

Rice is an important food crop in China, which not only carries the heavy responsibility of food production, but also has wetland ecosystem functions such as pollutant retention and pollution control, which is of great significance to the production of food security and maintenance of farmland ecosystem stability [1]. The co-culture system of rice and eco-friendly aquatic animals such as crabs, fish, shrimps, frogs and ducks has become more and more a focus of attention, especially the rice-crab co-culture composite ecosystem. The rice-crab aquaculture model builds an agroecosystem that is mutually reinforcing and interdependent. Rice provides crabs with a cool environment and abundant food

---

Biographies: Yang Guo, research interest: theory and technology of water saving irrigation in arid areas, Email: nxuguoyang2024@163.com.

*Corresponding author: Juncang Tian, Professor, research interest: theory and technology of water saving irrigation in arid areas. School of Civil and Hydraulic Engineering, Ningxia University, No.539 Helanshan West Road, Xixia District, Yinchuan 750021, China. Tel: +86-13995112538, Email: slxtjc@163.com.

resources needed for growth, and the activities of crabs not only contribute to soil loosening and weed control in rice paddies, but also their excreta can be used as natural fertilizers for rice, effectively reducing the use of chemical fertilizers and pesticides. This naturally cycling agroecosystem not only effectively increases soil nutrient concentration, enzyme activity, soil microbial biomass carbon, and microbial biomass nitrogen [2], as well as more efficient nitrogen use and reduced nutrient loss [3], but also greatly reduces the negative impacts of agricultural production on the environment [4], including the use of chemical pesticides and fertilizers. In addition, crabs, as a high value-added agricultural product, were added to the rice-crab symbiosis system, which improved land utilization and output efficiency, and gradually developed into a "low-carbon and high-efficiency recycling aquaculture model" characteristic of the Yinbei Irrigation District.

As the cornerstones of the rice-crab ecosystem, soil and water are the most critical components of this complex system. Among them, the microbial community plays an irreplaceable role by closely linking the two ecosystems, soil and water, with its unique way of existence [5]. Microorganisms of the soil influence the growth, quality and yield of rice by decomposing the organic matter in the soil, participating in the mineralization process, and influencing the utilization of soil nutrients and the biogeochemical cycling of elements [6]. In rice agroecosystems, the pelagic microbiota reflects the condition of the water source and the surrounding soil [7]. In addition, the microbial community has the important function of regulating the water environment and realizing water purification, which together with rice and crabs constitute a harmonious ecosystem.

Current research on rice-crab symbiotic systems focuses on rice yield, soil physicochemical properties, efficiency of crop production, and greenhouse gas emissions [43], [44], however, relatively few studies have been conducted on the distribution and cycling of microorganisms in the overall ecosystem. It is noteworthy that maintaining the diversity of microbial species is crucial for building sustainable agricultural production systems [8]. Microorganisms, especially fungi, in soil and water bodies play crucial roles in nutrient transformation, pollutant degradation, and crop disease prevention [9]. Therefore, enhancing the study of microbial communities in rice-crab symbiosis system can help us to understand the functioning mechanism of this ecosystem more comprehensively. In this experiment, we performed microbial sequencing based on the Illumina sequencing platform on rice inter-root soil, soil at the bottom of the ring ditch, water samples from the ring ditch, and water samples from the paddy field in the rice-crab aquaculture area, and analyzed the abundance, composition, and diversity of bacterial and fungal diversity at different sites in the rice-crab symbiotic environment in order to determine the distribution of bacterial and fungal functional microorganisms in the rice-crab symbiotic aquaculture system, and to promote healthier agro-agricultural aquaculture provide a solid foundation for the promotion of healthy agricultural aquaculture.

1 Materials and methods

1.1 [illegible]erimental design

The experimental area is situated in Helan County, Yinchuan City, Ningxia Hui Autonomous Region, China (38°40′30″N, 106°17′56″E). The soil texture of the test area is irrigation silt soil, and the irrigation water source is Yellow River water. The altitude of the test site is 1102 m. The dry bulk density of the soil in the tillage layer (40 cm) is 1.43 g·cm^{-3}, and the water holding rate in the field is 22.5%. The basic soil properties included soil pH of 8.46, organic matter of 9.45 mg·kg^{-1}, alkaline dissolved nitrogen of 32.2 mg·kg^{-1}, effective phosphorus of 11.6 mg·kg^{-1}, and quick-acting potassium of 128.0 mg·kg^{-1}.

The rice variety was QiuYou 88, transplanted on May 25, 2018 and harvested on October 8th. Crab seedlings were brought in on May 28th and harvested on September 5th. The plots were 170 m long and 40 m wide, with row and plant spacing of 10 cm × 5 cm, and a 30-cm-high anti-climbing net was set at the periphery. Rice was harvested at maturity ([illegible]ber 1st). Samples were collected on September 4, 2021, rice inter-root soil (RS), soil at the bottom of ring ditch (DS), ring ditch water sample (SW) and paddy water sample (PW), each sample was collected at three locations and mixed and set in three replicates. Soil and water samples were cooled, immediately transported to the laboratory and stored at -20°C for further analysis.

1.2 DNA extraction and high-throughput sequencing

Total DNA from soil samples was extracted using the Mobio Soil Microbial DNA Power Extraction Kit PowerSoil DNA [illegible]ation Kit. Bacterial 16S rDNA (V3+V4) regional primers 5'-ACTCCTACGGGGAGGCAGCA-3' and 5'-GGACTACHVGGGTWTCTAAT-3' were used to amplify the target fragments of the samples. Fungi were amplified using universal primers ITS2 gene (5'-TAGAGGAAGTAAAAGTCGTAA-3' and 5'-TTCAAAGATTCGATGATTCAC-3'). The fragments obtained by amplification were purified and recovered by 1% agarose gel electrophoresis, and then library construction and sequencing were performed by Shanghai Majorbio Bio-pharm Technology Co.,Ltd

1.3 Serial processing

Multiplexed decomposition of raw sequencing reads with adjusted splices and quality. Sequences were initially denoised to remove sequences containing sequencing errors. The reads of each sample were assembled using FLASH v1.2.7 through overlap, and the resulting assembled sequences were considered as raw Tags data. The raw Tags obtained from the assembly process were then filtered using Trimmomatic v0.33 to acquire high-quality Tags data. Chimeric sequences were identified and removed using UCHIME v4.2, yielding the ultimate valid data. The Tags were clustered using UCLUST in QIIME (version 1.8.0) software at 97% similarity level to obtain OTUs, and the OTUs were taxonomically annotated based on the Silva (Bacteria) taxonomic database. Diversity indices (Ace,

Chao, Shannon, Simp-son) were calculated using Muthur software and clustered for analysis.

1.4 Data processing

Relevant data processing and statistical analysis were performed using Excel 2010 and SPSS 19.0 software. Plots were made using Origin 2021b, and the significance of differences was tested at the P<0.05 level using the LSD method.

2. Results and analysis

2.1 Analysis of sequencing results

The high-throughput sequencing results are shown in Tab 1, 12 bacterial samples obtained a total of 639,489 pairs of Reads, filtered to produce a total of 574,663 Clean tags, producing an average of 47,888 Clean tags and an average sequence length of 415 bp. A total of 1138709 pairs of Reads were obtained from 12 fungal samples, and a total of 989,311 Clean tags were generated after filtering, with an average of 82,442 Clean tags and an average sequence length of 218 bp. As shown in Table 1, the number of OTUs of the four groups of samples was 447 ~ 4336. the Chao index was 282 ~ 4102, and the Shannon index was 3.16 ~ 6.86. all the samples had a coverage greater than or equal to 0.97, which indicated that the results of the present sequencing represented the real situation of the microorganisms in the samples [10]. According to the richness indices (Chao1 and Ace), the bacterial richness was DS > RS > PW > SW in descending order, and the diversity indices (Shannon and Invsimpson) followed the same trend. Fungal diversity was lower than bacterial diversity and richness was DS > RS > PW > SW in descending order.

Tab 1 Number of taxonomic units and diversity index for each group of samples

	Sample	OTUs	Shannon	Simpson	Ace	Chao1	Coverage
Bacteria	DS	4336	6.86±0.07	0.003±0.0002	4133.65±108.24	4101.86±86.32	0.97±0.0004
	RS	4043	6.74±0.14	0.004±0.0012	3763.25±382.15	3767.00±339.62	0.973±0.003
	PW	2078	4.51±0.03	0.073±0.0082	2655.83±180.8	2058.57±111.40	0.98±0.0010
	SW	1601	4.14±0.16	0.101±0.0191	2140.02±24.39	1729.46±56.26	0.99±0.0004
Fungi	DS	903	3.70±0.18	0.07±0.02	742.55±71.19	730.31±73.39	0.997±0.0004
	RS	881	3.56±0.66	0.12±0.10	525.71±115.37	525.08±106.17	0.999±0.0003
	PW	668	3.35±0.21	0.08±0.02	413.53±65.64	417.44±66.07	0.999±0.0001
	SW	447	3.16±0.08	0.09±0.01	278.26±49.42	282.48±53.19	1.000±0.0003

Analysis by principal coordinate analysis (PCoA) based on the Bray-Curtis measure of variance is an important tool for assessing overall differences in microbial community structure. PCoA plots showed aggregation of both bacterial and fungal communities by sample origin (Figure 1). According to the PCoA analysis, the first two principal coordinates of bacterial PCoA, PC1 and PC2, explained 88.7% and 7.17% of the total variation. In contrast, the first and second axes explained similar variations in the fungal communities, which together accounted for 81.48% of the total variance, with both having a p-value of 0.001.

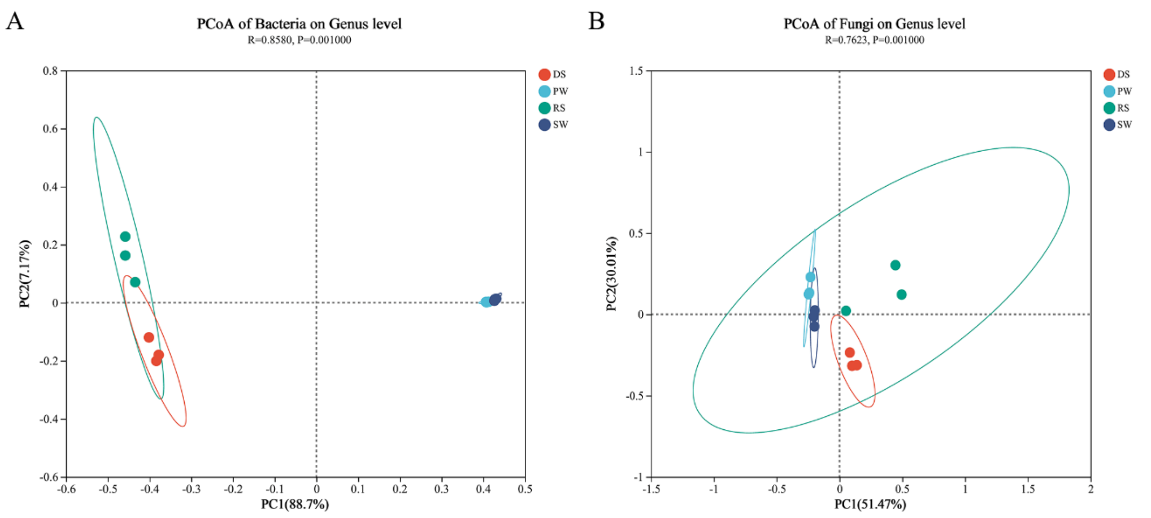

Fig 1 Principal coordinate analysis presenting spatial variation in bacterial and fungal communities according to Bray-Curtis distance (A: Bacterial, B: Fungal)

2.2 Composition of microbial community structure

A total of 54 bacterial phyla were identified at the phylum level across the four sample groups (Fig 3), among which 14 phyla had an abundance of >1%. Considering phyla with an abundance of >10% as dominant, the bacterial community compositions of DS and RS were relatively similar, with Proteobacteria, Bacteroidetes, Actinobacteria, Chloroflexi and Desulfobacterota as the dominant phyla. Compared to the RS, the DS showed a decrease in the relative abundance of Actinobacteria, Chloroflexi, Acidobacteria and Gemmatimonadetes by 6.15%, 7.85%, 2.01%, and 1.74%, respectively, while the proportions of other phyla increased. Notably, the relative abundances of Bacteroidetes and Desulfobacterota increased significantly by 7.4% and 4.9%, respectively. Similarly, the bacterial community compositions of PW and SW were also comparable, with Proteobacteria, Actinobacteria, Bacteroidetes and Verrucomicrobia as the dominant phyla. In comparison to the SW, the PW exhibited a decline in the relative abundance of Cyanobacteria, Actinobacteria, Verrucomicrobia and Vibrionaceae by 5.91%, 0.47%, 1.59%, and 0.21%, respectively, while the proportions of other phyla increased. Notably, Proteobacteria and Bacteroidetes showed significant increases of 4.88% and 1.77%, respectively.

A total of 1227 genera were identified from the bacterial samples of the four groups at the genus level, with 49 genera having an abundance of >1%. The dominant genera were defined as those accounting for more than 5% of the total. The bacterial community compositions of DS and RS were relatively similar, but neither had a dominant genus exceeding 5%. In the DS, the main genera included *norank_f_Bacteroidetes_vadinHA17* (4.06%), *norank_f_steroidobacteraceae* (2.49%), *norank_f_Prolixibacteraceae* (2.36%) and *norank_f_norank_o_SBR1031* (2.11%). In contrast, the RS was dominated by *norank_f_norank_o_SBR1031* (3.97%), *norank_f_norank_o_norank_c_KD4-96*

(2.97%), *Marmoricola* (2.95%), *norank_f_Anaerolineaceae* (2.59%), *Nocardioides* (2.39%), *Thiobacillus* (2.20%), *norank_f_norank_o_Vicinamibacterales* (2.14%) and *norank_f_norank_o_Ardenticatenales* (2.04%). Distinct from the DS and RS, the major dominant bacteria in the bacterial community composition of PW and SW were *norank _f_norank_o_chloroplast* (27.75%~33.05%) and *Rhodobacteracea* (5.49%~6.89%). Additionally, the relative abundance of relatively abundant genera such as *LD29*, *Cyanobium_PCC-6307*, *norank_f_MWH-UniP1_aquatic_group*, *Hydrogenophaga* and *hgcl_clade* were all greater than 2%.

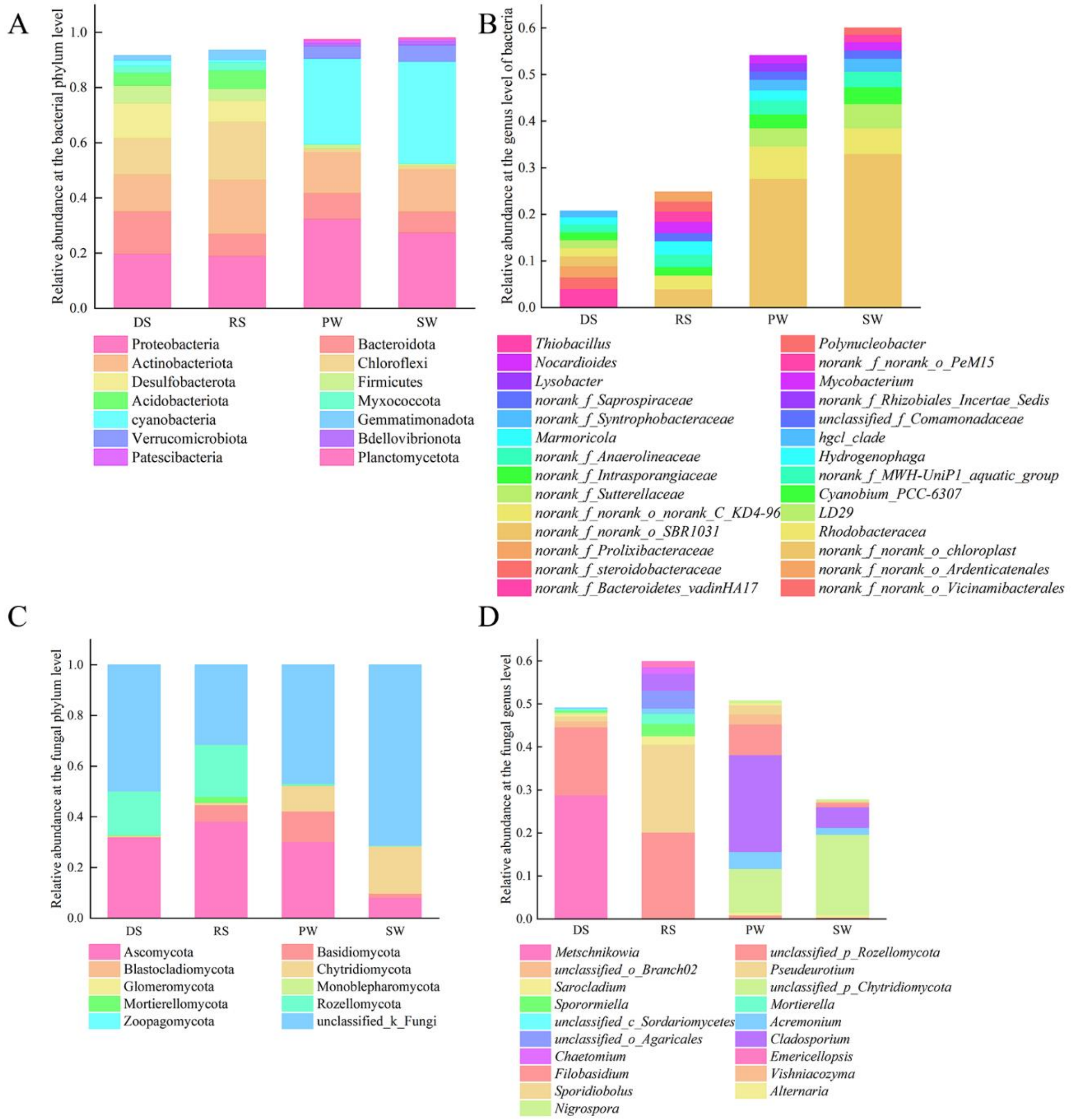

Fig 2 Bacteria and fungi at the phylum and genus level Fungi in the top 10 of the total (A: Bacteria phylum level, B: Bacterial genus level, C: Fungal phylum level, D: Fungal genus level)

Fungal diversity was relatively low in all samples compared to bacteria, with only nine phyla detected in all samples (Fig 2), as well as a large number of identified fungal phyla. DS and RS fungal

communities were relatively similar in composition, with the dominant phyla being Ascomycota and Rozellomycota, and the relative abundance of Stramonium, Ascomycota, Rozellomycota, Monohaematococcus and Potamonium decreased in the DS compared to the RS by 6.41, 6.17, 3.23, 1.97, and 0.45%, respectively, while the proportion of the remaining phyla changed by less than 0.02%. The composition of fungal communities in the PW and SW was similar, with the dominant phyla being Ascomycetes, Staphylococcus and Puccinomycetes, and the relative abundance of Ascomycetes and Staphylococcus increased by 21.98% and 10.57%, respectively, while that of Puccinomycetes decreased by 8.57% in comparison with that of the SW.

A total of 97 genera were identified at the genus level for the fungi of the four groups of samples, as well as (31.46%-71.32%) unidentified fungal genera. As shown in Fig. 4, there was a difference in the genus level distribution of the fungal communities of the 4 groups of samples. *unclassified_p_Rozellomycota* (15.86%~20.18%) was the co-dominant genus in DS and RS, in addition to possessing the dominant genera of *Metschnikowia* (28.81%) and *Pseudeurotium* (20.45%), respectively. *unclassified_p_Chytridiomycota* (10.09%-18.66%) was the co-dominant genus in PW and SW, in addition to which *Filobasidium* (7.12%) was detected as the dominant genus in PW.

2.3 LEfSe differential significance analysis

The different biomarkers for LDA with LDA scores >4 in the linear discriminant effects analysis (LEfSe) are shown in Figure 3. These biomarkers showed significant variation in the relative abundance of their core microorganisms and were accompanied by considerable variation in response to environmental disturbances. The 36 bacterial evolutionary branches showed significant differences in all samples. Samples from DS, RS, PW, and SW were enriched with different bacteria, with only 3, 8, 2, and 5 abundant bacterial evolutionary branches, respectively.

At the genus level, DS soils are mainly rich in *Steroidobacteraceae*, *Prolixibacteraceae* and *Bacteroidetes_vadinHA17*. RS is mainly rich in *Thiobacillus*, *Vicinamibacterales*, *Marmoricola*, *Nocardioides*, *Anaerolineaceae*, SBR1031, *Ardenticatenales* and *KD4-96*. PW was concentrated in *Rhodobacieraceae*, *Hydrogenophaga*, while SW was *MWH-UniP1_aquatic_group*, *Chloroplast*, *PCC-6307*, *hgcl_clade*, *LD20*, and its most important taxon was Chloroplast in the Cyanobacteria (LDA score > 5.0). The results of the LEfSe analysis were generally consistent with the results of the microbial community analysis above. Overall, there were significant differences in microbial composition between sampling sites, which to some extent reflected the influence of the overall circulating microbial community.

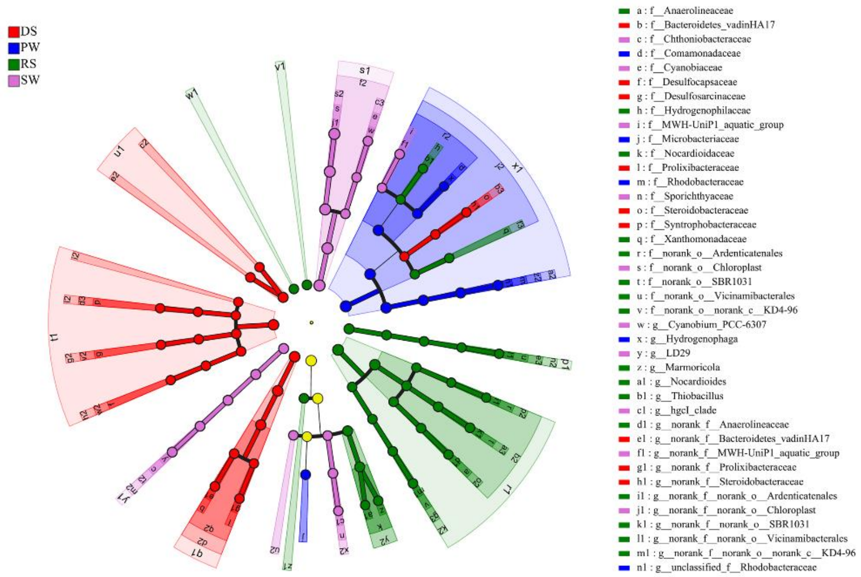

Fig 3 LEfSe analysis of the microbial communities of the samples

(Circles radiating from inside to outside represent hierarchical categories from phylum to genus, each small circle at different taxonomic levels represents the category at that level, and the small diameter circle's represent relative abundance. Nodes of different colors indicate microbiota significantly enriched in the corresponding group, and yellow nodes indicate microbiota with no significant differences)

2.4 Prediction of functional genes of soil bacteria

The results of FAPROTAX functional predictions are shown in Fig 4. The major taxa were all chlorophyll, chemoheterotrophy, aerobic chemoheterotrophy, aerobic nitrite oxidation, nitrification, ureolysis, aromatic compound degradation, and hydrocarbon degradation. One-third of the metabolic functions had a relative abundance greater than 1%. The bacterial OTUs of DS and RS were mainly enriched in aerobic chemical heterotrophy, fermentation, aromatic compound degradation, nitrate reduction, sulfur compound respiration, dark oxidation of sulfur compounds, dark sulfide oxidation, sulfate respiration, nitrate respiration, and nitrogen respiration, which were significantly higher than those of PW and SW ($P<0.05$). PW and SW possessed richer functional ecological bacteria of chloroplasts, phototrophs, photo-autotrophs, cyanobacteria, and aerobic photo-autotrophs. In addition to this, small amounts of lignin decomposition, aromatic hydrocarbon degradation, aliphatic non-methane hydrocarbon degradation, xylan decomposition, and cellulose decomposition (abundance < 2%) were identified in all samples used.

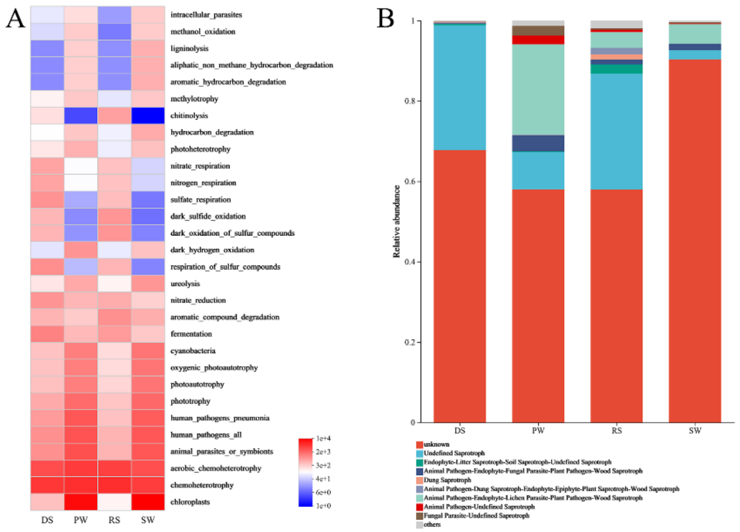

Fig 4 Bacterial and fungal functional gene prediction (A: Bacterial, B: Fungal)

A total of 296 OUTs were annotated with trophic pattern functional information after removing unclassified OTUs by FUNGuild prediction (Fig 4). The trophic patterns of fungi in all four groups of samples were dominated by saprotrophs, undefined saprotrophs and Animal Pathogen-Endophyte-Lichen Parasite-Plant Pathogen-Wood Saprotroph, which accounted for 32.78% of the OTUs of the four groups of samples, respectively, 31.16%, 31.81% and 7.11%, respectively. The symbiotic pattern group was mainly composed of clumped mycorrhizae, endophytes and ectomycorrhizae. RS identified the highest number of functional groups. Among them Endophyte-Litter Saprotroph-Soil Saprotroph-Undefined Saprotroph (2.30%), Animal Pathogen-Dung Saprotroph-Endophyte-Epiphyte-Plant Saprotroph-Wood Saprotroph (1.61%), and Dung Saprotroph (1.26%) were higher than the other three groups of samples. While Animal Pathogen-Endophyte-Fungal Parasite-Plant Pathogen-Wood Saprotroph (4.01%), Animal Pathogen-Undefined Saprotroph (2.16%) and Fungal Parasite-Undefined Saprotroph (2.43%) were more prevalent in the PW than in the other three groups.

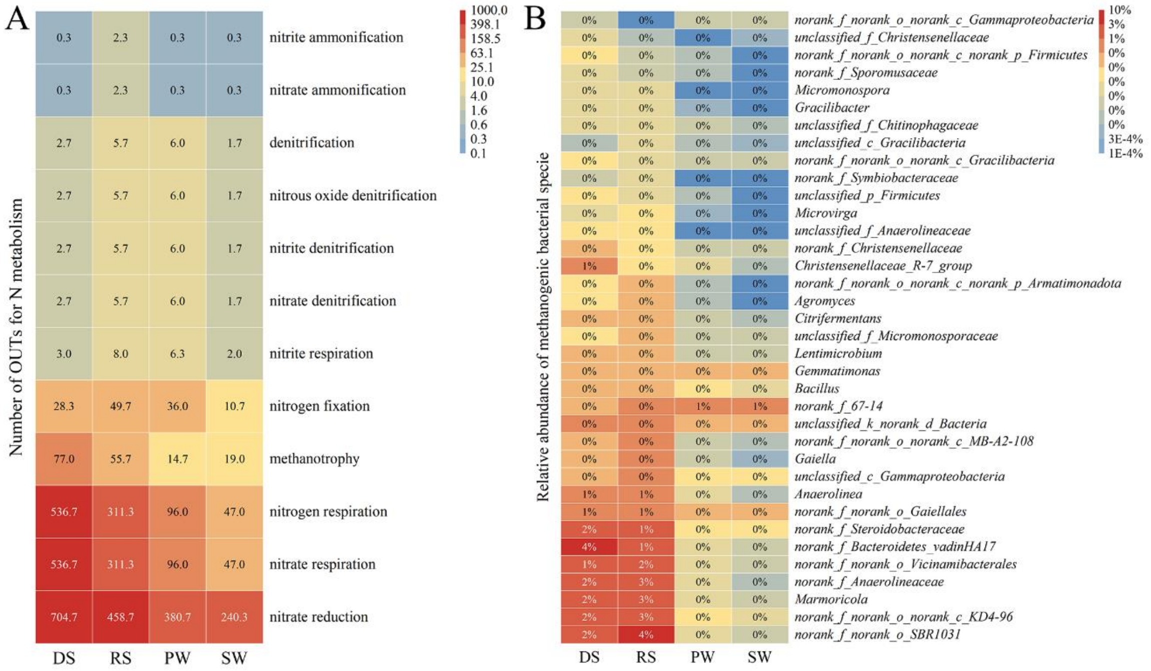

Fig 5 Number of OUTs for N metabolism and relative abundance of methanogenic bacterial species (A: N metabolism, B: methanogenic)

The number of OUTs associated with the N cycle by FAPROTAX annotation is shown in Fig 5. These include nitrogen fixation, nitrate respiration, N-respiration, nitrous oxide denitrification, denitrification, nitrate respiration, nitrate denitrification, and nitrite denitrification. The number of OTUs was ranked as DS>RS>PW>SW. The relative abundance of methanogenic species was ranked RS (22.60%) > DS (19.79%) > SW (1.62%) > PW (1.61%).

2.5 Co-occurrence network analysis

In order to reveal potential interactions between bacteria, the whole microbiome network of microbial associations between the four groups of samples, respectively, Spearman correlations between OTUs were computed based on their patterns of occurrence in the samples to better understand the coexistence of the microbial communities (Fig 6). In total, 50 nodes were significantly correlated in bacteria, while 48 pairs of nodes were strongly correlated in fungal samples. Rhizobiales, Comamonadaceae, Ardenticatenales, Algoriphagus, and Novosphingobium were important in the bacterial correlation network. Gibberella, Hannaella, Papiliotrema, Gibellulopsis played an important role in the fungal correlation network (Tab 2).

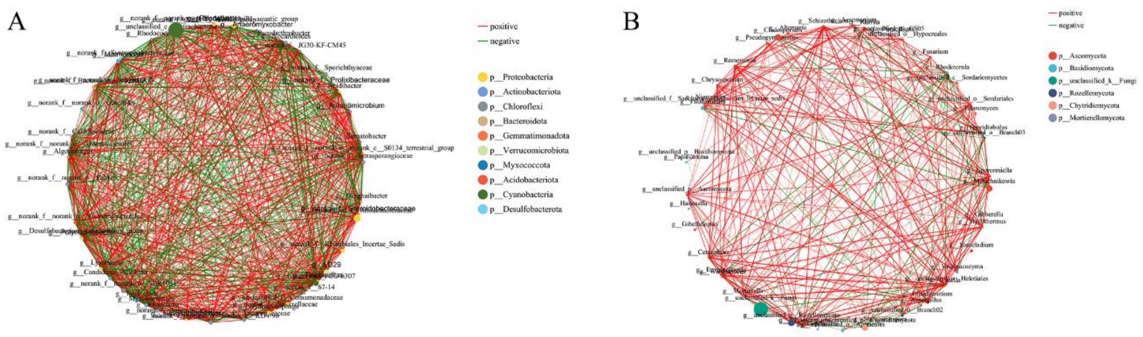

Fig 6 Species-species correlations plotted on a network diagram (A: Bacterial, B: Fungal)

Table 2 Attributes of the top 15 network nodes (left: bacteria; right: fungi)

Node	Degree	Clustering coefficient	Node	Degree	Clustering coefficient
g_norank_f_Rhizobiales_Incertae_Sedis	38	0.933	*g_Gibberella*	3	1.000
g_unclassified_f_Comamonadaceae	40	0.927	*g_Hannaella*	4	1.000
g_norank_f_norank_o_Ardenticatenales	41	0.924	*g_Papiliotrema*	2	1.000
g_Algoriphagus	39	0.920	*g_Gibellulopsis*	5	0.900
g_Novosphingobium	40	0.914	*g_Aspergillus*	8	0.893
g_unclassified_f_Rhodobacteraceae	40	0.914	*g_Chaetomium*	21	0.857
g_Acidibacter	42	0.912	*g_unclassified_o_GS05*	11	0.855
g_Candidatus_Aquiluna	41	0.907	*g_Chrysosporium*	19	0.842
g_norank_f_norank_o_PeM15	41	0.907	*g_unclassified_k__Fungi*	18	0.837
g_Mycobacterium	41	0.907	*g_Kernia*	19	0.830
g_norank_f_norank_o_Vicinamibacterales	42	0.906	*g_unclassified_o_Branch03*	14	0.791
g_Aurantimicrobium	38	0.895	*g_unclassified_o_Helotiales*	20	0.789
g_norank_f_Caldilineaceae	41	0.890	*g_Fusarium*	16	0.783
g_Hydrogenophaga	40	0.890	*g_Pseudeurotium*	23	0.767
g_Rhodobacter	41	0.884	*g_Emericellopsis*	23	0.767

3. Discussion

3.1 Characterization of community structure and functional composition at the portal level

The phylum Ascomycetes, Actinobacteria, Mycobacteria and Green Benders were found to be the most abundant phylum in most of the samples in this study, similar to the study of LIU [42]. Most of the Ascomycetes are trophic, with relatively fast growth rate and ability to use a variety of substrates, and are common bacterial phylum in rice fields [11]. Actinomycetes act on organic matter in the environment mainly through the production of hydrolytic enzymes, help maintain the biological balance of the soil by cooperating with nutrient cycling, and play an important role in the recycling of organic carbon [12]. The phylum Green Benders, the first dominant phylum in RS, is classified as phototrophic and non-phototrophic, and has a non-negligible role in soil carbon cycling by utilizing unstable carbon in the environment, degrading starch, sugars, and peptides, and providing organic acids to other organisms in the sediment [13]. Desulfovibrio, Acidobacteria and Thick-walled Bacteria were the dominant phyla in the soil, but their abundance was substantially reduced in the water samples.

Cyanobacteria were the dominant phylum in water samples.

Fungal diversity results showed that Ascomycota was the most abundant phylum in all samples. The abundance of Rozellomycota in soil was significantly greater than its abundance in water bodies. Rozellomycota are mostly found in freshwater, soil or marine ecosystems, often found in phytopathogenic fungi and prefer to consume soil organic matter [36]. In addition to this, it may play an important role in the phosphorus cycle [14]. Diversity at the PW gate level was significantly greater in water samples than in SW. The relative abundance of Chytridiomycota was greater than 10% in all water samples. Chytridiomycota is a common aquatic fungus and many species under its phylum are saprophytic and active in water-saturated (oxygenated) paddy soils. It is also known to be a parasite that infects a variety of phytoplankton, zooplankton, fungi, invertebrates and plants [37]. In general, a certain number of parasitic fungal phyla affecting the healthy growth of incoming crops are present in both soil and water bodies.

3.2 Characterization of community structure and functional composition at the genus level

Functionally speaking, chemoheterotrophy, aerobic chemoheterotrophy and phototrophic autotrophy functional groups constitute the primary energy sources for the ecological soil prokaryotic community in the rice-crab symbiotic system. In the paddy soil and surrounding ditch soil, inorganic nitrogen fixation is dominant, while organic nitrogen consumption prevails. In contrast, photosynthetic autotrophy is responsible for carbon fixation in paddy water and surrounding ditch water, as evidenced by the abundant presence of chloroplasts. Rice cultivation is an important source of total agricultural greenhouse gas emissions. In this study, we identified a large number of bacteria closely related to methanogenic bacterial species. The relative abundance of *norank-f-Bacteroidetes-vadinHA17* was 4.06% in DS, which was significantly higher than the other treatments ($p<0.05$), and was a sawdust and polyacrylamide degrader [15]. The relative abundance of *norank_o_SBR*1031 was 3.98% and 2.11% in RS and DS, respectively, which has been less reported in studies, and is a dominant genus in the cooling phase of the composting process and in the maturation phase, which may contribute to the conversion of nitrogenous compounds [20]. *norank_f_steroidobacteraceae* is positively correlated with soil NO_3-N concentration [18] and participates in carbon and nitrogen cycling by degrading organic matter and consuming organic acids for denitrification and methane production [17]. It is noteworthy that there are also some genera with relative abundances ranging from 1% to 2% in the soil samples. The *norank_c_KD4-96* within the Chloroflexi phylum can enhance the relative abundance of Fe(II) oxidizing groups, such as *Gallionellaceae* and *Nitrospira*, which possess the capabilities of Fe absorption and nitrite oxidation [21]. Furthermore, *Gemmatimonas* and *Sporomusaceae* play pivotal roles in the degradation of phenanthrene and the production of CH_4 in paddy fields [16].

Microorganisms involved in carbohydrate degradation were found in abundance in all soil samples.

In the bacterial fraction. *norank_c_Dojkabacteria*, *norank_f_Prolixibacteraceae* and *Smithella* are members of the acid/acetic acid producing population that ferment polysaccharides (cellobiose and starch) into acids, which can have an effect on environmental pH [19]. *Marmoricola* is a Gram-positive bacterium, widely found in marble, beach sediments and forest soils, etc., which assimilates a variety of sugars and organic compounds such as D-glucose, phenylacetic acid, adipic acid and biphenyl [22], [23]. A strain of *Marmoricola* isolated from wheat was found to degrade a fungal toxin, Deoxynivalenol (DON), which affects the growth of cereals (rice, maize, wheat, etc.). *Nocardioides* (2.39%) is a highly efficient genus for degrading DON, utilizing DON under aerobic conditions as carbon and sole source of energy, this strain degrades DON to produce the new intermediate 3-epi-DON [24]. It is noteworthy that the genus *Nocardioides*, which has a degrading function for herbicides, was distributed in multiple samples [25], RS (2.386%), DS (1.168%), PW (0.0466%), and SW (0.009532%). Bacteria of the genus *Nocardioides* can utilize a wide range of organic substances as a carbon source, including petroleum hydrocarbons, aromatic compounds, and azaheterocyclic compounds, and can also effectively perform steroid biodegradation and biotransformation [27]. In addition carbohydrate fermenting bacteria *norank_f_Anaerolineaceae* were found in soil, which have the ability to degrade carbonaceous organic matter to CO_2 [26].

In the fungi fraction. An unclassified strain of *unclassified_p_Rozellomycota* was found to be able to break down a large amount of biomass such as cow dung and straw being broken down into small molecules of monosaccharides or small amino acids all of which can be very good for providing nutrients to rice, as evidenced by the dung fungus *Sporormiella*. *Pseudeurotium* is a common peatland fungus and many species within the genus have strong cellulolytic activity. It was found that biochar (rice, wheat, corn stover, etc.) can increase the relative abundance of potentially beneficial fungi, but too much biochar is rather unfavorable [38]. In addition to this, the genus *Pseudeurotium* is known to promote crop growth and inhibit pathogenic bacteria, as well as degrade volatile organic compounds [39]. *Pseudeurotium* is more common in traditional monocropped rice soils and its abundance is higher in soil samples free of heavy metal contamination, while its abundance decreases in soils heavily contaminated with heavy metals [40].

In water samples, *norank_f_norank_o_Chloroplast* and *unclassified_f_Rhodobacteraceae* were found to be the dominant genera in PW and SW. There are fewer studies on *norank_o_Chloroplast*, Qiu found it to be the dominant genus of cultured substrate, which was positively correlated with the environmental factor TAN, and had no correlation with TP and TN [29], and Yu et al. analyzed indicated that it was a nitrogen-fixing bacterium [30]. *Rhodobacteraceae* are aquatic bacteria, including aerobic photo and chemoheterotrophs and purple non-sulfur bacteria that photosynthesize in anaerobic environments [31]. *Rhodobacteraceae* are characterized by the photosynthesis gene cluster (PGC), which encodes all proteins of anaerobic photosynthesis, enzymes of the bacterial chlorophyll and carotenoid biosynthesis pathways, regulatory proteins and cofactors [32]. LD26 is a highly abundant

genus in water samples and a dominant genus in SW, which is affiliated with Spartobacteria. macrogenomic analysis revealed that LD26 possesses the ability to utilize carbon sources common in phytoplankton (cellulose, mannan, xylan, chitin, starch) and sulfated polysaccharides [33]. *Cyanobium PCC-6307* is a freshwater cyanobacterium commonly found in warm water, which acts as an indicator taxon in the water for possible bloom pollution [34]. In addition, the photosynthetic bacterial cyanobacteria in the water body provide energy to the water environment of the ring ditch and paddy field, but there is also a risk that the abundance of cyanobacteria in the water body is at a high level, which needs to be alerted to the occurrence of water bloom.

In addition to functional probiotics, pathogenic bacteria are also an important part of the microorganisms in the rice-crab symbiotic system. *Cladosporium delicatulum* of the genus Cladosporium and *Acremonium stromaticum* of the genus Acremonium are pathogenic bacteria that infest rice grains, while the antagonistic bacterium Acremonium has antimicrobial, cytotoxic, phytotoxic, antiviral, insecticidal, and enzyme inhibitory activities [41], and the presence of both of them is required to maintain a smooth ecological functioning of the rice field. presence is required to maintain smooth ecological functioning of paddy fields. *Metschnikowia bicuspidata* belongs to the family Spermophtoraceae and was mainly concentrated in the ditch bottom soil of the present experimental field (28.81%), whereas its abundance in the rice soil was significantly lower (0.22%). *Metschnikowia bicuspidata* causes milky mildew disease in mitten crabs [28], resulting in wasting of the crab body and lower vigor. It causes wasting of the crab body, weaker vigor, opaque milky white coloration of the cephalothorax muscle tissue and joint membrane junctions at the base of the stepping foot, gradual reduction of hemolymph, decreased blood cohesion, and ultimately tissue or organ failure and death [35], and it can infect other aquatic organisms, such as roe shrimp, fish, and so on.

4. Conclusion

Rice growth and photosynthetic characteristics based on rice-crab symbiotic system and microbiological analysis of rice soil, ditch bottom soil, paddy water body, and ring ditch water body showed that higher chlorophyll and photosynthesis during the rice reproductive period showed significant differences in bacterial and fungal diversity between soil and water samples, whereas there was no significant difference in the diversity of the two soils versus the two water bodies. The dominant species at the bacterial phylum level were Ascomycetes, Cyanobacteria, Actinobacteria, Mycobacteria and Green Benders. The dominant species at the genus level were *norank_f_norank_o_Chloroplast*, *unclassified_f_Rhodobacteraceae*, *LD29*, *Cyanobium_PCC-6307* and *norank_f_MWH-UniP1_aquatic_group*. The dominant species at the fungal phylum water were unclassified_k_Fungi, Ascomycota, Rozellomycota, Potentilla, and Stramonium. The dominant species at the genus level were *unclassified_k_Fungi*, *unclassified_p_Rozellomycota*, *Metschnikowia*, *Cladosporium* and

unclassified_p_Chytridiomycota. After functional gene prediction, the dominant phylum may rely on mechanisms such as organic matter catabolism, secondary metabolite secretion, and photo-energetic autotrophs to adapt to and influence geologic habitats, and the main functional genes are all related to metabolic functions, such as secondary product metabolism, energy metabolism, and amino acid metabolism. Meanwhile, strains of the genera *Metschnikowia* and *Nocardioides* with the ability to influence the biochemical environment of the rice-crab symbiotic system have been discovered and focused on, which will provide the necessary support base for the healthy development of agricultural aquaculture.

Reference

[1] Wu W, Ma B, Uphoff N (2015) A review of the system of rice intensification in China. Plant and soil 393: 361-381.

[2] Bashir MA, Wang H, Pan J, Khoshnevisan B, Sun W, et al. (2021) Variations in soil nutrient dynamics and their composition in rice under integrated rice-crab co-culture system. Journal of cleaner production 281: 125222.

[3] Hu L, Ren W, Tang J, Li N, Zhang J, et al. (2013) The productivity of traditional rice–fish co-culture can be increased without increasing nitrogen loss to the environment. Agriculture, Ecosystems and Environment 177: 28-34.

[4] Li M, Li R, Zhang J, Wu T, Liu S, et al. (2020) Effects of the integration of mixed-cropping and rice–duck co-culture on rice yield and soil nutrients in southern China. Journal of the Science of Food and Agriculture 100(1): 277-286.

[5] Zhou R, Hou D, Zeng S, Wei D, Yu L, et al. (2022) Sedimentary nitrogen and sulfur reduction functional-couplings interplay with the microbial community of anthropogenic shrimp culture pond ecosystem. Frontiers in Microbiology 13: 830777.

[6] Oyewole OA (2012) Microbial Communities and Their Activities in Paddy Fields: a Review. Journal of Veterinary Advances 2(2): 74-80.

[7] Reche MHLR, Fiuza LM (2005) Bacterial diversity in rice-field water in Rio Grande do Sul. Brazilian Journal of Microbiology 36: 253-257.

[8] Yadav AN, Kour D, Rana KL, Kumar V, Dhaliwa SV, et al. (2017) Plant microbiomes and its beneficial multifunctional plant growth promoting attributes. International Journal of Environmental Sciences and Natural Resources 3(1): 01-08.

[9] Prasad S, Malav LC, Choudhary J, Kannojiya S, Kundu M, et al. (2021) Soil microbiomes for healthy nutrient recycling. Current trends in microbial biotechnology for sustainable agriculture 1: 21.

[10] Caporaso JG, Lauber CL, Walters WA, Berg-Lyons D, Lozupone CA, et al. (2011) Global patterns of 16S rRNA diversity at a depth of millions of sequences per sample. Proceedings of the national academy of sciences 108(supplement_1): 4516-4522.

[11] Wu M, Qin H, Chen Z, Wu J, Wei W (2011) Effect of long-term fertilization on bacterial composition in rice paddy soil. Biology and Fertility of Soils 47: 397-405.

[12] Bhatti AA, Haq S, Bhat RA (2017) Actinomycetes benefaction role in soil and plant health. Microbial pathogenesis 111: 458-467.

[13] Hug LA, Castelle CJ, Wrighton KC, Thomas BC, Sharon I, et al. (2013) Community genomic analyses constrain the distribution of metabolic traits across the Chloroflexi phylum and indicate roles in sediment carbon cycling. Microbiome 1: 1-17.

[14] Ng CWW, Liao JX, Lau SY, San So P, Hau BCH, et al. (2023) Coupled effects of elevated CO_2 and biochar on microbial communities of vegetated soil. Journal of Environmental Management 342: 118136.

[15] Hao L, Liu Y, Chen N, Hao X, Zhang B, et al. (2021) Microbial removal of vanadium (V) from groundwater by sawdust used as a sole carbon source. Science of the Total Environment 751: 142161.

[16] Wang YQ, Wang MX, Chen YY, Li CM, Zhou ZF (2021) Microbial community structure and co-occurrence are essential for methanogenesis and its contribution to phenanthrene degradation in paddy soil. Journal of Hazardous Materials 417: 126086.

[17] Jang S W, Yoou M H, Hong W J, Kim YJ, Lee EJ, et al. (2020) Re-Analysis of 16S Amplicon Sequencing Data Reveals Soil Microbial Population Shifts in Rice Fields under Drought Condition. Rice 13: 1-7.

[18] Wu AL, Jiao XY, Wang JS, Dong EW, Guo J, et al. (2021) Sorghum rhizosphere effects reduced soil bacterial

diversity by recruiting specific bacterial species under low nitrogen stress. Science of The Total Environment 770: 144742.

[19] Zhao J, Li Y, Euverink GJW (2022) Effect of bioaugmentation combined with activated charcoal on the mitigation of volatile fatty acids inhibition during anaerobic digestion. Chemical Engineering Journal 428: 131015.

[20] Lei L, Gu J, Wang X, Song Z, Wang J, et al. (2021) Microbial succession and molecular ecological networks response to the addition of superphosphate and phosphogypsum during swine manure composting. Journal of Environmental Management 279: 111560.

[21] Wang J, Ma Y, Di L, Qian X, Wang G (2021) Straw incorporation with nitrogen amendment shapes bacterial community structure in an iron-rich paddy soil by altering nitrogen reserves. Microorganisms 9(5): 988.

[22] Kim S J, Lim J M, Hamada M, Ahn JH, Weon HY, et al. (2015) *Marmoricola solisilvae* sp. nov. and *Marmoricola terrae* sp. nov., isolated from soil and emended description of the genus Marmoricola. International Journal of Systematic and Evolutionary Microbiology 65(Pt_6): 1825-1830.

[23] Li J, Peng K, Zhang D, Luo C, Cai X, et al. (2020) Autochthonous bioaugmentation with non-direct degraders: a new strategy to enhance wastewater bioremediation performance. Environment international 136: 105473.

[24] Ikunaga Y, Sato I, Grond S, Numaziri N, Yoshida S, et al. (2011) *Nocardioides* sp. strain WSN05-2, isolated from a wheat field, degrades deoxynivalenol, producing the novel intermediate 3-epi-deoxynivalenol. Applied microbiology and biotechnology 89: 419-427.

[25] Harada N, Takagi K, Harazono A, Fujii K, Iwasaki A (2006) Isolation and characterization of microorganisms capable of hydrolysing the herbicide mefenacet. Soil Biology and Biochemistry 38(1): 173-179..

[26] Tang Z, Lin Z, Wang Y, Zhao P, Kuang F, et al. (2020) Coupling of thermophilic biofilm-based systems and ozonation for enhanced organics removal from high-temperature pulping wastewater: Performance, microbial communities, and pollutant transformations. Science of the Total Environment 714: 136802.

[27] Ma Y, Wang J, Liu Y, Wang X, Zhang B, et al. (2023) *Nocardioides*: "specialists" for hard-to-degrade pollutants in the environment. Molecules 28(21): 7433.

[28] Jiang H, Cao G, Bao J, Feng C, Li X, et al. (2024) Effect of temperature on the occurrence of *Metschnikowia bicuspidata* disease in *Eriocheir sinensis*. Aquaculture 585: 740695.

[29] Qiu CW, Shi YH, Wang HX (2021) Microbial community structure in different cultural patterns of *Takifugu obscures* [in Chinese]. Journal of Fisheries of China 45(02):209-220.

[30] Yu X, Yu K, Liao Z, Liang J, Deng C, et al. (2020) Potential molecular traits underlying environmental tolerance of *Pavona decussata* and *Acropora pruinosa* in Weizhou Island, northern South China Sea. Marine Pollution Bulletin 156: 111199.

[31] Pujalte MJ, Lucena T, Ruvira MA, Arahal DR, Macián MC (2014) The family rhodobacteraceae. Springer.

[32] Brinkmann H, Göker M, Koblížek M, Wagner-Döbler I, Petersen J (2018) Horizontal operon transfer, plasmids, and the evolution of photosynthesis in *Rhodobacteraceae*. The ISME journal 12(8): 1994-2010.

[33] Mohapatra M, Behera P, Kim JY, Rastogi G (2020) Seasonal and spatial dynamics of bacterioplankton communities in a brackish water coastal lagoon. Science of the Total Environment 705: 134729.

[34] Hongxia M, Jingfeng F, Jiwen L, Zhiyi W, Yantao W, et al. (2021) Full-length 16S rRNA gene sequencing reveals spatiotemporal dynamics of bacterial community in a heavily polluted estuary, China. Environmental Pollution 275: 116567.

[35] Bao J, Jiang H, Shen H, Xing Y, Feng C, et al. (2021) First description of milky disease in the Chinese mitten crab Eriocheir sinensis caused by the yeast *Metschnikowia bicuspidata*. Aquaculture 532: 735984.

[36] Zhang ZF, Pan YP, Liu Y, Li M (2021) High-level diversity of basal fungal lineages and the control of fungal community assembly by stochastic processes in mangrove sediments. Applied and Environmental Microbiology 87(17): e00928-21.

[37] Blaalid R, Khomich M (2021) Current knowledge of Chytridiomycota diversity in Northern Europe and future research needs. Fungal Biology Reviews 36: 42-51.

[38] He X, Xie H, Gao D, Khashi U RahmanM, et al. (2021) Biochar and intercropping with potato–onion enhanced the growth and yield advantages of tomato by regulating the soil properties, nutrient uptake, and soil microbial community. Frontiers in Microbiology 12: 695447.

[39] Zhou X, Shen Y, Fu X, Wu F (2018) Application of sodium silicate enhances cucumber resistance to Fusarium wilt and alters soil microbial communities. Frontiers in plant science 9: 624.

[40] Zheng X, Song K, Li S, Zhang H, Bai N, et al. (2021) Effects of long-term integrated agri-aquaculture on the soil fungal community structure and function in vegetable fields. Scientific Reports 11(1): 10813.

[41] Tian J, Lai D, Zhou L (2017) Secondary metabolites from Acremonium fungi: Diverse structures and bioactivities. Mini Reviews in Medicinal Chemistry 17(7): 603-632.

[42] Liu GL, Niu SW, Xu JY, Sui SS, Ye X, et al. (2019) Effects of optimized n fertilization on rice soil bacterial diversity of rice crab symbiosis system analyzed by high-throughput sequencing technology [in Chinese]. Journal of Jilin Agricultural University 41(06):686-694.

[43] Wang A, Ma X, Xu J, Lu, W (2019) Methane and nitrous oxide emissions in rice-crab culture systems of northeast China. Aquaculture and Fisheries 4(4): 134-141.

[44] Bashir MA, Wang H, Pan J, Khoshnevisan B, Sun W.et al. (2021) Variations in soil nutrient dynamics and their composition in rice under integrated rice-crab co-culture system. Journal of cleaner production 281: 125222.
