# Supplementary material for: Composition and functional diversity of soil and water microbial communities in the rice-crab symbiosis system
Source: PLoS One. 2025 Jan 17;20(1):e0316815. doi: 10.1371/journal.pone.0316815 (PMC11741653; doi:10.1371/journal.pone.0316815)
Supplement: Attachment — Submitted filename: Response to Reviewers1216.docx [file pone.0316815.s004.docx]

# Response to Reviewers

Dear Sayed Haidar Abbas Raza,

I hope this email finds you well.

Thank you very much for your thorough review and valuable feedback on my submitted article titled " Composition and functional diversity of soil and water microbial communities in the rice-crab symbiosis system". Upon receiving your comments, I promptly embarked on revising and enhancing the article based on your suggestions. Below are the specific adjustments I have made and some additional notes:

**Format and Style:**

I have adjusted the article's layout according to the formatting guidelines you provided, including heading levels, paragraph spacing, font size, etc., to meet publication standards. I have checked and supplemented the complete form of the OTU in the article.

**Images and Citations:**

I have made a chart showing the rice crab ecosystem and the areas where the samples were collected. As per your request, I have replaced some images to ensure all are copyright-clear. I have uploaded the graphic files to the Preflight Analysis and Conversion Engine (PACE) and ensured that the graphics meet the requirements.

**Data：**

All data I have added to the attachment is free.

**References：**

For all cited literature, I have verified and updated the latest publication information.

Thank you once again for your meticulous guidance and support! I look forward to our next collaboration and hope to receive your further feedback soon.

Wishing you all the best and good health!

Best regards,

Guo Yang

Email: nxuguoyang2024@163.com

Date: December 16, 2024
